# Chemotherapy plus Panitumumab Versus Chemotherapy plus Bevacizumab in Metastatic Colorectal Cancer: A Meta-analysis

**DOI:** 10.1038/s41598-017-19001-6

**Published:** 2018-01-11

**Authors:** Zhigui Li, Yuqian Huang, Rui Zhao, Yaping Cui, Yong Zhou, Xiaoting Wu

**Affiliations:** Department of gastrointestinal surgery, West China Hospital, Sichuan University, Chengdu, Sichuan 610041 China

## Abstract

Panitumumab and bevacizumab have been widely used in combination with chemotherapy for patients with wild type RAS metastatic colorectal cancer (mCRC). Whether panitumumab or bevacizumab was the optimal option remained controversial. Thus, we conducted a meta-anaylsis to evaluate chemotherapy plus panitumumab (C + P) versus chemotherapy plus bevacizumab (C + B) in wild type RAS mCRC. Electronic databases including PubMed, Embase, and Web of Science, Cochrane Library, ClinicalTrials.gov, were searched. This meta-analysis estimated the progression-free survival (PFS), overall survival (OS), overall response rate (ORR) and adverse events (AEs). Three randomized controlled trials with a total number of 577 patients were included. In wild type RAS population, PFS [hazard ratio (HR) = 0.96; 95% confidence interval (CI), 0.76 to 1.15] and OS (HR = 0.90; 95% CI, 0.54 to 1.27) and ORR [relative ratio (RR) = 2.06; 95% CI, 0.86 to 4.90] appeared similar between the two treatments, the incidence of AEs slightly increased (RR = 1.16; 95% CI 1.08 to 1.26). In conclusion, there was insufficient evidence to precisely conclude that combination treatment of C + P had an improved efficacy compared with C + B. Further large-scale and better-designed clinical trials are still needed to evaluate the combination treatment of C + P in patients with wild type RAS mCRC.

## Introduction

All over the world, more than one million patients were diagnosed annually with colorectal cancer, one of the most common causes of cancer-related mortality^1^. Approximately 15–25% of patients with colorectal cancer had metastatic disease at the time of diagnosis, up to 50% of all patients would develop metastases which commonly occurred in the liver or lung^2^. The five-year relative survival rate was only 5–15% in patients with metastatic colorectal cancer (mCRC). During the recent decades, the mortality from mCRC has declined on account of improving earlier detection and advances in comprehensive treatment, especially in chemotherapy combined with targeted monoclonal antibodies; the median overall survival (OS) time increased from approximately one year to two years or more.

Chemotherapy combined with targeted monoclonal antibodies were the most principal therapeutic approaches in patients with mCRC^3–6^. Two chemotherapy regimens (FOLFOX and FOLFIRI) were usually considered to be the first-line treatment options in these patients. Except from adverse events (AEs), the two regimens were similar in terms of OS, progression-free survival (PFS), overall response rate (ORR)^7,8^; thus,choice of targeted monoclonal antibodies have attracted more and more attention. Bevacizumab is a targeted monoclonal antibody against vascular endothelial growth factor (VEGF). Compared with chemotherapy alone, combination treatment of chemotherapy plus bevacizumab (C + B) has been shown to improve outcomes^5^. Several clinical trials and guidelines around the world suggested that combination treatment of C + B was an option for first-line treatment of mCRC^9,10^. Panitumumab is an immunoglobulin G monoclonal antibody against epidermal growth factor receptor (EGFR). Similarly, combination treatment of chemotherapy plus panitumumab (C + P) has been shown to be superior to chemotherapy alone^11^. It was observed that mCRC patients with mutation of RAS had no beneficial effects of anti-EGFR (cetuximab and panitumumab) treatment^12,13^. Therefore, RAS gene status was a predictive biomarker for the effects of anti-EGFR treatment in mCRC^14,15^. For anti-VEGF treatment, it was unnecessary to test RAS gene status. Previous study^16^ demonstrated that the addition of anti-EGFR to chemotherapy significantly improved OS compared with C + B; there was more heterogeneity because of anti-EGFR drug including panitumumab and cetuximab. In contrast, two trials reported no significant improvement on OS and PFS between C + P and C + B^17,18^.

Whether combination treatment of C + P was superior to combination treatment of C + B remained controversial. The aim of this meta-analysis was to evaluate the efficacy and safety of C + P versus C + B in wild type RAS mCRC according to the major clinical trials.

## Results

**Figure 1:**
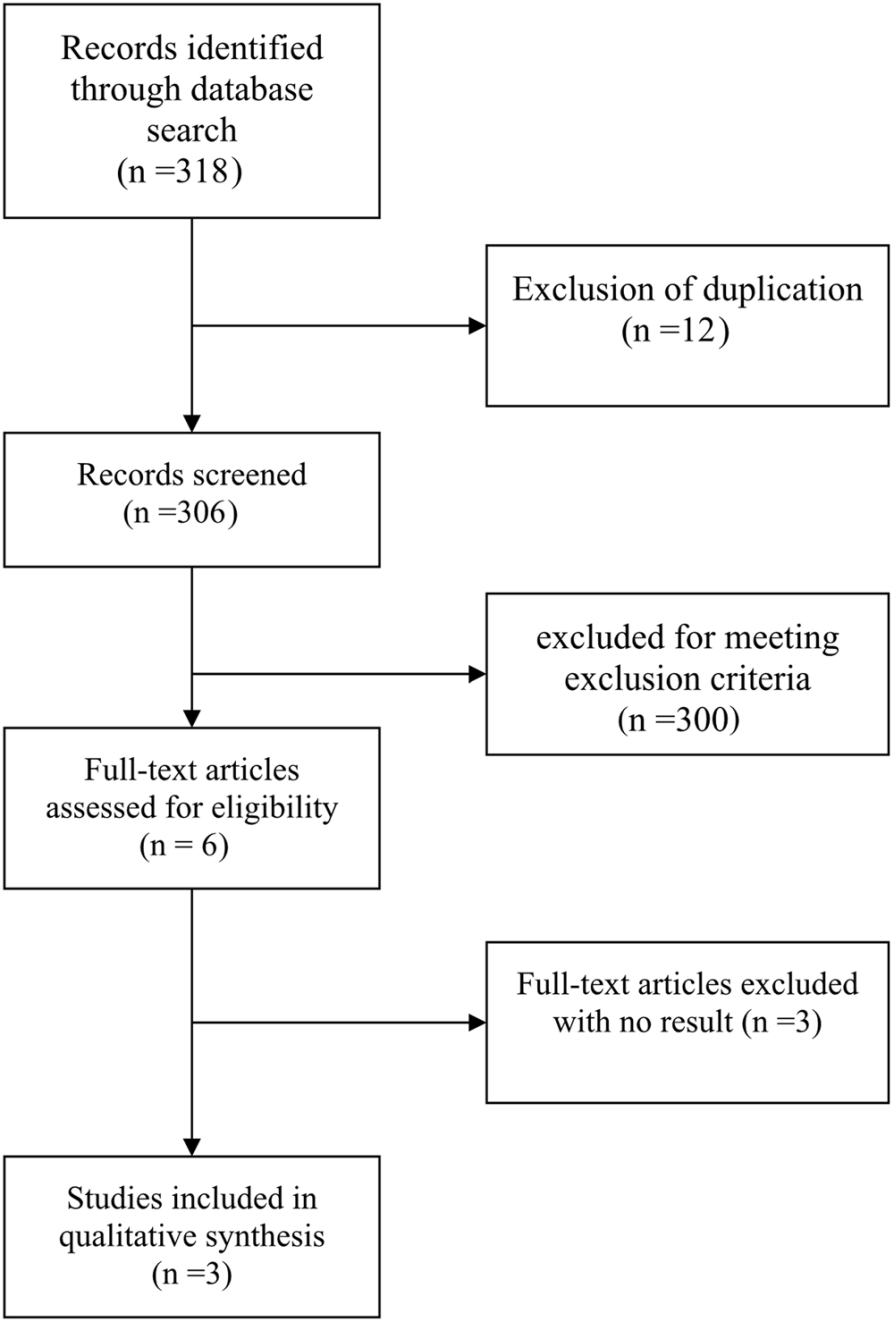
Flow chart showing literature search and study selection.

The research procedure was presented in Fig. 1. The title and abstract of 319 studies were reviewed. After the initial screening, 313 studies were excluded in accordance with our exclusion criteria. For the full-text information evaluation, we reviewed the remaining six studies on comparing C + P versus C + B, which were published between 2014 and 2017, three randomized clinical trials were exluded in meta-analysis because of no results^19–21^. The rest of three suitable trials were PEAK (comparing panitumumab + mFOLFOX6 versus bevacizumab + mFOLFOX6, ClinicalTrials.gov Identifier: NCT00819780)^22^, SPIRITT (comparing panitumumab + FOLFIRI versus bevacizumab + FOLFIRI, ClinicalTrials.gov Identifier: NCT00418938)^17^, WJOG 6201 G (comparing panitumumab + FOLFIRI versus bevacizumab + FOLFIRI Clinical, UMIN Clinical Trials Registry: UMIN000005216)^18^. These trials included a total of 577 patients with wild type RAS exon 2 mCRC, which comprised 278, 182, 117 patients from PEAK, SPIRITT, WJOG 6201 G, respectively. This table (Table 1) showed the characteristics of these clinical trials. According to the latest guidelines in the Cochrane Handbook for Systematic Reviews of Interventions, we employed methodological quality assessment and deemed the quality of the included studies was high (data not shown).

**Table 1:** Baseline characteristics of patients in the trials included in the meta-analysis.

Authors	Study	Year	Country	Treatment regimen	Sample size	Age	KRAS test	Primary end point	Line of treatment
Schwartzberg *et al*.	PEAK	2014	USA	Panitumumab(6 mg/kg)-mFOLFOX6 Bevacizumab(5 mg/kg)-mFOLFOX6	139/139	63(23, 82) 61(28, 82)	exon2	PFS	First-line therapy
Hecht, J. R *et al*.	SPIRITT	2015	mutlticenter	Panitumumab(6 mg/kg)- FOLFIRI Bevacizumab(5 or 10 mg/kg)- FOLFIRI	91/91	60(27, 84) 60(25, 80)	exon2	PFS	Second-line therapy
Kohei Shitara *et al*.	WJOG 6210 G	2016	Japan	Panitumumab(6 mg/kg)- FOLFIRI Bevacizumab(5 mg/kg)- FOLFIRI	59/58	62(31, 82) 64(26, 78)	exon2	OS	Second-line therapy

**Figure 2:**
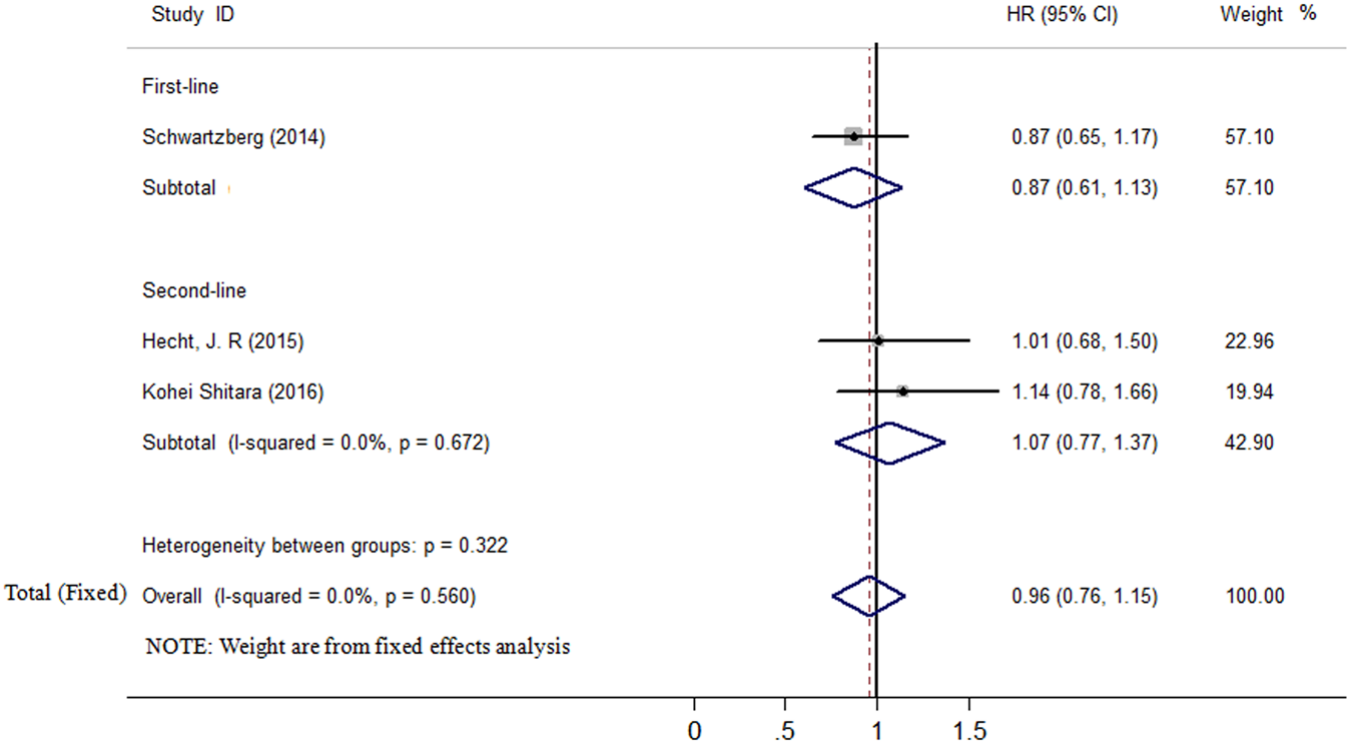
Forest plot showing meta-analysis results of progression-free survival.

All the included studies reported the data of PFS, OS, ORR, AEs. The median PFS among these studies ranged from 6.0 to 10.9 months in the panitumumab group, and from 5.9 to 10.1 months in the bevacizumab group, respectively. Because no significant heterogeneity was found (*I*^2^ = 0.0%, *P* = 0.560), the fixed-effects model analysis was performed to explore the pooled results. In the PFS analysis, the hazard ratio (HR) for C + P versus C + B was 0.96 [95% confidence interval (CI) 0.76 to 1.15], indicating no statistically significant difference between two treatments. In subgroup analysis based on line of treatment, the HR for C + P versus C + B was 0.87 (95% CI 0.61 to 1.13) in first-line treatment, 1.07 (95% CI 0.77 to 1.37) in second-line treatment (Fig. 2).

**Figure 3:**
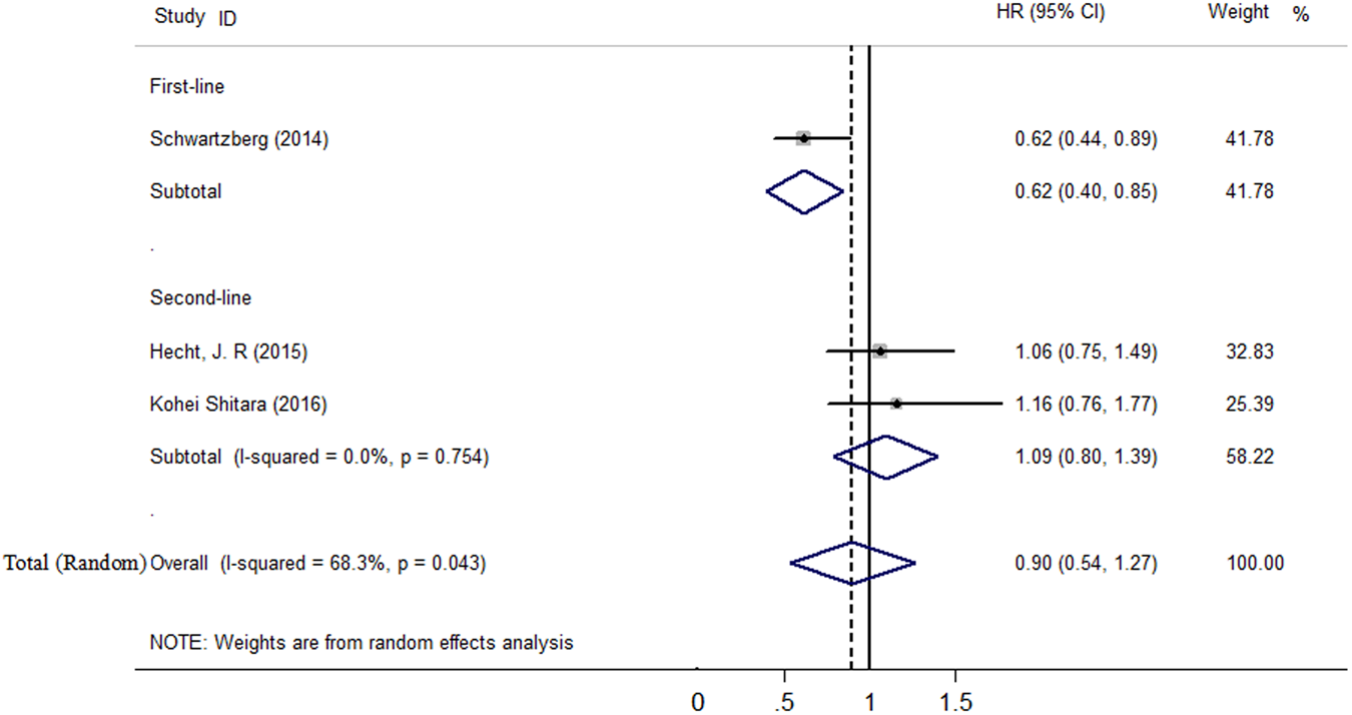
Forest plot showing meta-analysis results of overall survival.

The median OS ranged from 16.2 to 34.2 months in the panitumumab group, and from 13.4 to 24.3 months in the bevacizumab group, respectively. Because there was significant heterogeneity (*I*^2^ = 68.3%, *P* = 0.043), we performed the random-effects model analysis to better explore the pooled results. In the OS analysis, the HR for C + P versus C + B was 0.90 (95% CI 0.54 to 1.27). We also performed sensitivity analysis and subgroup analysis to explore the heterogeneity. When PEAK trial was removed, the heterogeneity was reduced; the pooled estimates did not substantially change. In subgroup analysis based on line of treatment, the HR for C + P versus C + B was 0.62 (95% CI 0.40 to 0.85) in first-line treatment, 1.09 (95% CI 0.80 to 1.39) in second-line treatment, respectively, indicating that OS was higher in patients who received combination treatment of C + P when it was used in first-line treatment (Fig. 3).

**Figure 4:**
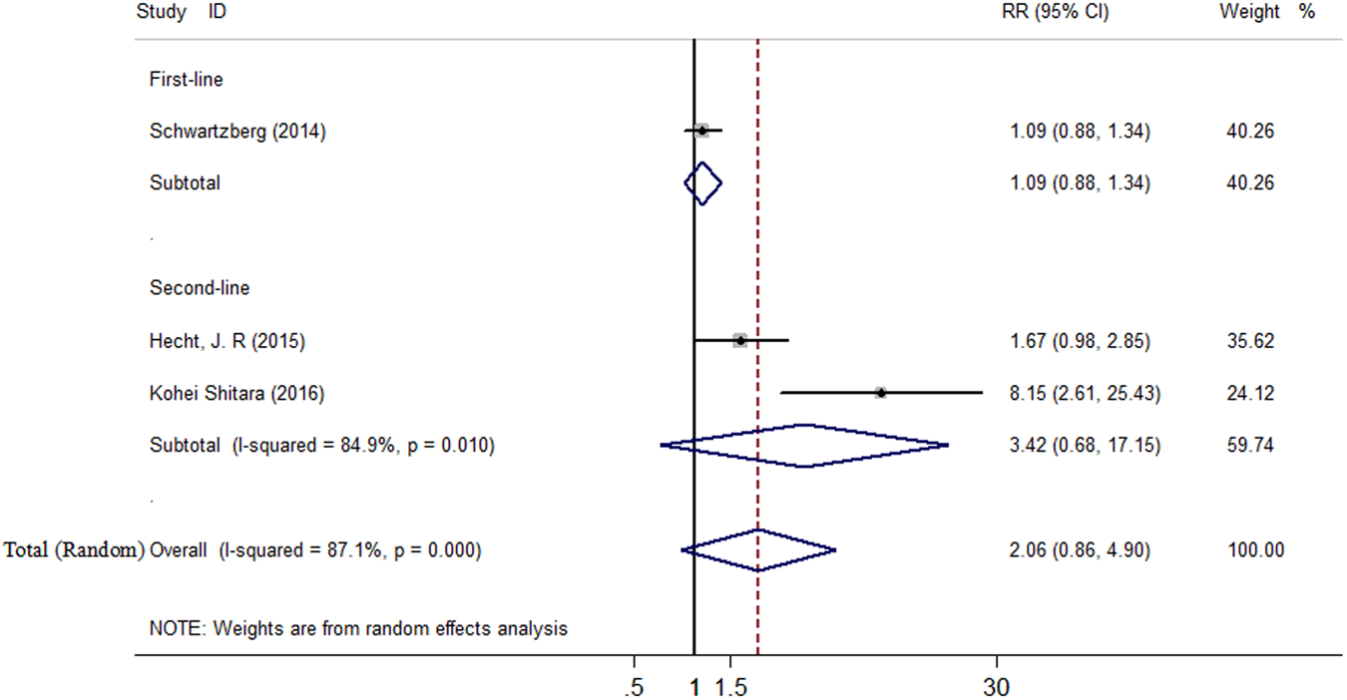
Forest plot showing meta-analysis results of overall response rate.

The ORR ranged from 32% to 57.8% in the panitumumab group, and from 5.7% to 53.5% in the bevacizumab group, respectively. Because significant heterogeneity was found (*I*^2^ = 87.1%, *P* = 0.000), the random-effects model analysis was performed to better explore the pooled results. In the ORR analysis, the relative ratio (RR) for C + P versus C + B was 2.06 (95% CI 0.86 to 4.90). We also performed sensitivity analysis and subgroup analysis. When excluding any single trial, the pooled estimates did not substantially change, significant heterogeneity was still present. In subgroup analysis based on line of treatment, the RR for C + P versus C + B was 1.09 (95% CI 0.88 to 1.34) in first-line treatment, 3.42 (95% CI 0.68 to 17.15) in second-line treatment, respectively (Fig. 4).

**Figure 5:**
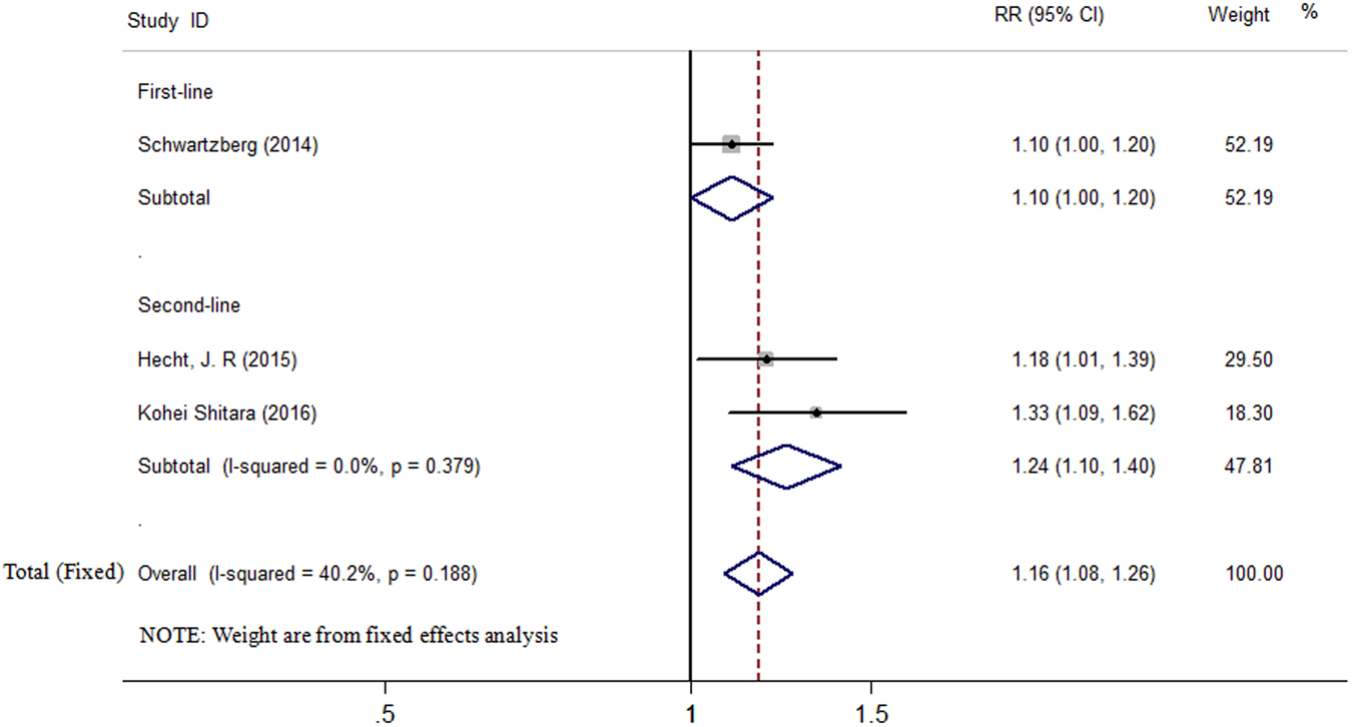
Forest plot showing meta-analysis results of adverse events.

Among the three included studies, the incidence of AEs ranged from 85% to 91% in the panitumumab group, and from 66.7% to 83% in the bevacizumab group, respectively. Because no significant heterogeneity was found (*I*^2^ = 40.2%, *P* = 0.188), the fixed-effects model analysis was performed to explore the pooled results. In the AEs analysis, the RR for C + P versus C + B was 1.16 (95% CI 1.08 to 1.26). We did not perform sensitivity analysis. In subgroup analysis based on line of treatment, the RR for C + P versus C + B was 1.10 (95% CI 1.00 to 1.20) in first-line treatment, 1.24 (95% CI 1.10 to 1.40) in second-line treatment, respectively (Fig. 5).

## Discussion

Previous studies have demonstrated that irinotecan-based (FOLFIRI) or oxaliplation-based chemotherapy (FOLFOX) has significantly increased survival in patients with mCRC. Until recently, several studies have shown that the combination of monoclonal antibodies against VEGF or EGFR with chemotherapy would improve clinical outcomes^3,23^. Some clinical trials and guidelines around the world suggested that combination treatment of C + B was an option in first-line treatment of mCRC^9,10^ and C + P was effective and well-tolerated in the treatment of patients with mCRC, especially in those with wild type RAS mCRC.

The present study was a meta-analysis aiming to investigate the efficacy and safety of C + P versus C + B in wild type RAS mCRC. Our review included the results of 3 studies, 1 in first-line treatment and 2 in second-line treatment, showed that the panitumumab group and bevacizumab group had similar results of PFS, OS and ORR. Although there was heterogeneity among these included trials, the overall consequences of the meta-analysis were consistent with a viewpoint that patients with wild type RAS mCRC would obtain a potential clinical benefit from combination treatment of C + P. In 2014, Schwartzberg, L. S. *et al*. published a randomized controlled trial (PEAK) aiming to evaluate the efficacy and safety of panitumumab plus mFOLFOX6 versus bevacizumab plus mFOLFOX6 in patients with wild type RAS mCRC. For wild type KRAS exon 2 analysis among PEAK study, the median PFS was 10.9 months in the panitumumab group, and 10.1 months in the bevacizumab group (*P* = 0.353), respectively; the median OS was 34.2 months in the panitumumab group, 24.3 months in the bevacizumab group (*P* = 0.009), respectively. After excluding patients with mutation in other RAS genes, the median PFS was 13.0 months in the panitumumab group, 9.5 months in the bevacizumab group (*P* = 0.029), respectively. The median OS was 41.3 months in the panitumumab group, 28.9 months in the bevacizumab group (*P* = 0.058), respectively. Between the two treatments, similar ORRs and AEs were observed. Patients undergoing AEs with a worst grade of ≥3 experienced more skin toxicity and hypomagnesemia in the panitumumab group, those experienced more hypertension in the bevacizumab group^24^.

SPIRITT study was a phase 2 trial to evaluate the efficacy and safety of panitumumab versus bevacizumab in the second-line combination treatment of wide type RAS mCRC. There was no significant difference in the PFS and OS and ORR. AEs (the worst grade ≥3) in the panitumumab group were prone to skin disorders, diarrhea, hypomagnesemia, hypokalemia, dehydration, and hypotension. Neutropenia was more frequent in the bevacizumab group.

WJOG 6210 G trial was a phase 2 trial with a total of 117 enrolled mCRC patients refractory to first-line chemotherapy containing oxaliplatin and bevacizumab. In the wild type KRAS exon 2 mCRC patient population, the median OS was 16.2 months in the panitumumab group, and 13.4 months in the bevacizumab group, respectively, but low power to detect differences. The median PFS was no significant difference between the two groups (HR, 1.14; 95% CI, 0.78–1.66). For all wide type RAS patients, the ORR was higher in the panitumumab group than in the bevacizumab group; whereas it was obvious lower in the panitumumab group than in the bevacizumab group among those with any RAS or BRAF mutations. In subgroup analysis, patients with low serum VEGF-A level had a better OS in the panitumumab group than in the bevacizumab group, but patients with high serum VEGF-A level had a better OS in the bevacizumab group than in the panitumumab group.

Two ongoing trials are designed to evaluate the efficacy and safety of C + P versus C + B in patients with wild type RAS mCRC^19,20^. PARADIGM study is designed to recruit a total of 800 patients from May 2015 to 2020 (ClinicalTrials.gov Identifier: NCT02394795) to investigate whether bevacizumab or panitumumab with standard chemotherapy (mFOLFOX6) is the optimal first-line treatment in patients with wild-type RAS mCRC. The primary endpoint is OS; secondary endpoints are PFS, response rate, duration of response, and curative resection rate. CRIRO 5 is a prospective, multicenter, randomized clinical trial registered at ClinicalTrials.gov (NCT02162563), and designed to investigate the optimal systemic induction therapy for mCRC patients with unresectable, liver-only metastases. All included patients will be tested for RAS mutation status. Patients with wild type RAS will be treated with chemotherapy (FOLFOX or FOLFIRI) plus bevacizumab or panitumumab, and patients with mutant RAS will be treated with chemotherapy (FOLFOX or FOLFIRI or FOLFOXIRI) plus bevacizumab. One trial (ClinicalTrials.gov Identifier: NCT01508000)^21^ has been terminated in September 2016 and no outcome was published because of low or poor accrual. We expect these outcomes will be published and update this meta-analysis.

In 2015, a systematic review and meta-analysis^25^ included seven eligible rondomized controlled trials^22,26–31^ and demonstrated that the addition of anti-EGFR (panitumumab and Cetuximab) to chemotherapy significantly improved OS, PFS and ORR compared with chemotherapy alone, the addition of anti-EGFR to chemotherapy significantly improved OS, but not PFS or ORR compared with C + B. In overall wild type RAS population analysis, anti-EGFR treatment significantly improved OS, PFS, ORR. In 2016, Heinemann, V. *et al*.^16^ published a systematic review including 3 studies^22,32,33^ and evaluating the clinical outcomes of anti-EGFR plus chemotherapy versus C + B, concluded that chemotherapy plus anti-EGFR or bevacizumab are effective first-line treatments for patients with wild type RAS mCRC. Because of anti-EGFR drug including panitumumab and Cetuximab, there was more heterogeneity. It has to be highlighted that our meta-analysis selectively evaluated the efficacy and safety of C + P versus C + B, provided more credible evidences which firmly support ESMO consensus conference^2,34^.

The detection of RAS mutation status before planning the therapeutic scheme of mCRC patients was clinical practice. It was widely recognized that mutations in KRAS, BRAF, NRAS, and PIK3CA are significantly associated with a low response rate^35^. Several recent studies have indicated that KRAS mutation is a negative predictive factor for panitumumab treatment so that the use of panitumumab or cetuximab had been restricted to patients without mutation of RAS.

This meta-analysis had several potential limitations that should be taken into account. First, this meta-analysis included only three clinical trials. The largest trial (PEAK) in favor of panitumumab accounted for 41.2% of the sample set; all of them had relatively small sample size which limit interpretation of these results. Second, substantial heterogeneity was present among the included trials in that characteristics of patients and treatment regimens and methods for RAS status test were diverse. Combination treatment in WJOG 6210 G and SPIRITT study was FOLFIRI plus monoclonal antibodies, combination treatment in PEAK study was mFOLFOX6 plus monoclonal antibodies. It was well known that first-line and second-line treatment differ in response and PFS. KRAS exon 2 was tested in all included patients; other RAS mutation (KRAS exon3, exon 4 and NRAS exon 3, exon 4, BRAF exon 15) confirmed as a negative predictor in anti-EGFR treatment, did not assessed in all patients. Third, most of these included trials were observational studies, the possibility of selection bias and unidentified confounders couldn’t be exluded entirely.

In conclusion, this systematic review and meta-analysis showed that the panitumumab group and bevacizumab group had similar efficacy. To date, there was insufficient evidence to precisely conclude that combination treatment of C + P had an improved efficacy compared with C + B. Even then, we proposed that combination treatment of C + P would be a promising option for patients with wild type RAS mCRC, especially for patients refractory to bevacizumab combined with chemotherapy. Nevertheless, further large-scale and better-designed clinical trials are still needed to investigate the combination treatment of C + P.

## Methods

### Search strategy

We conducted a comprehensive literature search in PubMed, Embase, Web of Science, Cochrane Systematic Reviews, Clinical Trials.gov database from inception to March 29, 2017. The following search terms were used: (((((“Sigmoid Neoplasms” [Mesh]) OR sigmoid cancer)) OR ((((“Colonic Neoplasms” [Mesh]) OR colon cancer)) OR ((rectal neoplasms) OR ((“Colorectal Neoplasms” [Mesh]) OR colorectal cancer))))) AND ((((Bevacizumab) OR “Bevacizumab” [Mesh])) AND ((panitumumab) OR “panitumumab” [Supplementary Concept])). Additionally, the references of related reviews and included trials were also manually checked to recognize other potentially eligible articles.

### Review strategy

The Endote bibliographic software (Endnote X7) was been used to establish an electronic database and expurgate duplicate or uncorrelated records. The full manuscripts of eligible studies were reviewed independently by two trained investigators (Zhigui Li, Rui Zhao). Information including characteristics of patients, treatment protocols, sample size and outcomes was extracted and inserted into an electronic database. Any divergencies between reviewers were resolved by consensus and discussion with other coauthors and corresponding author.

### Inclusion and exclusion criteria

All clinical trials evaluating the efficacy and safety of C + P versus C + B for mCRC was considered eligible for meta-analysis. The following inclusion criteria were applied: patients with mCRC were certainly verified by histology or cytology and were treated with C + P or C + B; trial data reported on PFS, OS, and ORR, and AEs, status of RAS gene.

The exclusion criteria were as follows: case report, reviews, clinical trial registration that have no result, experimental animal studies. When several papers repetitively reported the same trial, we chose the most informative article for analysis.

### Data extraction and statistical analysis

Two trained independent investigators extracted the following data from eligible articles: main authors, published year, sample size and characteristics of patients, status of RAS gene, line of treatment, treatment regimens, median duration or hazard ratio with 95% CI of PFS and OS, ORR, AEs.

*I*^2^ statistics was performed to assess the heterogeneity between trials. If a little heterogeneity *(I*^2^ < 50%) was observed, the fixed-effects model analysis (Mantel-Haenszel method) was used to pool the estimates. If substantial heterogeneity *(I*^2^ ≥ 50%) was observed, the random-effects model (Mantel-Haenszel method) was applied for analysis. Sensitivity analysis or subgroup analysis was performed to explore the potential sources of heterogeneity. All statistical analyses were performed using STATA 12.0 software (Stata Corporation, College Station, TX, USA), and two-sided *P* value of less than 0.05 was considered statistically significant.

## Electronic supplementary material

supplementary information
