# Supplementary material for: Chemotherapy plus Panitumumab Versus Chemotherapy plus Bevacizumab in Metastatic Colorectal Cancer: A Meta-analysis
Source: Sci Rep. 2018 Jan 11;8:510. doi: 10.1038/s41598-017-19001-6 (PMC5764984; doi:10.1038/s41598-017-19001-6)
Supplement: Supplementary file 1 — supplementary information [file 41598_2017_19001_MOESM1_ESM.doc]

Title: Chemotherapy plus Panitumumab Versus Chemotherapy plus Bevacizumab in Metastatic Colorectal Cancer: A Meta-analysis

Authors list: Zhigui Li, Yuqian Huang, Rui Zhao, Yaping Cui, Yong Zhou, Xiaoting Wu

Address: Department of gastrointestinal surgery, West China Hospital, Sichuan University, Chengdu, Sichuan, 610041, China.

Corresponding author: Xiaoting Wu, MD, FACS, Department of gastrointestinal surgery, West China Hospital, Sichuan University, Chengdu, Sichuan, 610041, China. e-mail:wxt1@medmail.com.cn

| Authors(year) | study | methods | Median PFS | Median OS | Adverse events. | ORR(CR+PR) |
| --- | --- | --- | --- | --- | --- | --- |
| Schwartzberg, et al (2014) | PEAK | Panitumumab-mFOLFOX6  Bevacizumab-mFOLFOX6 | 10.9m (CI9.4 to 13.0)  10.1 m (CI,9.0 to 12.6)  (HR, 0.87;95%CI,0.65–1.17 | 34.2 m (CI26.6 m to not reached) 24.3 m (CI, 21.0 to 29.2m )  (HR, 0.62;CI,044–0.89) | 91%(126/139)  83%(115/139) | 57.8%(82/142)  53.5%(76/143) |
| Hecht, J. R, et al ( 2015) | SPIRITT | Panitumumab- FOLFIRI  Bevacizumab- FOLFIRI | 7.7 m(CI 5.7-11.8)  9.2 m (CI 7.8-10.6)  (HR 1.01,CI0.68-1.50) | 18.0 m (CI 13.5-21.7)  21.4 m (CI 16.5-24.6)  (HR 1.06,CI0.75-1.49). | 85%(77/91)  71%(65/91) | 32%(28/87)  19%(16/83) |
| Kohei Shitara, et al (2016) | WJOG 6210G | Panitumumab- FOLFIRI  Bevacizumab- FOLFIRI | 6.0m vs 5.9m (HR, 1.14;  95% CI, 0.78–1.66) | 16.2m  13.4m  (HR, 1.16; 95% CI, 0.76–1.77) | 88.5%(54/61)  66.7%(40/60) | 46.2%（24/52）5.7%(3/53) |

supplementary table 1:

abbreviation: m=month,CI=95% confidence interval
